# Seabird nutrients increase coral calcification rates and boost reef carbonate production

**DOI:** 10.1038/s41598-024-76759-2

**Published:** 2024-10-22

**Authors:** Ines D. Lange, Cassandra E. Benkwitt

**Affiliations:** 1 University of Exeter, Exeter, UK; 2 Lancaster Environment Centre, Lancaster University, Lancaster, UK

**Keywords:** Seabird nutrients, Coral growth, Coral calcification, Reef carbonate budget, Marine biology, Biogeochemistry, Ecosystem ecology, Ecosystem services, Tropical ecology

## Abstract

While excessive anthropogenic nutrient loads are harmful to coral reefs, natural nutrient flows can boost coral growth and reef functions. Here we investigate if seabird-derived nutrient subsidies benefit the growth of two dominant corals on lagoonal reefs, submassive *Isopora palifera* and corymbose *Acropora vermiculata*, and if enhanced colony-level calcification rates can increase reef-scale carbonate production. *I. palifera* and *A. vermiculata* colonies close to an island with high seabird densities displayed 1.4 and 3.2-times higher linear extension rates, 1.8 and 3.9-times faster planar area increase, and 1.6 and 2.7-times higher calcification rates compared to colonies close to a nearby island with low seabird densities, respectively. While benthic ReefBudget surveys in combination with average coral growth rates did not indicate differences in reef-scale carbonate production across sites, coral carbonate production was 2.2-times higher at the seabird-rich island when using site-specific linear growth rates and skeletal densities. This study shows that seabird-derived nutrients benefit fast-growing branching as well as previously unstudied submassive coral taxa. It also demonstrates that nutrient subsidies benefit colony-scale and reef-scale calcification rates, which underpin important geo-ecological reef functions. Restoring natural nutrient pathways should thus be a priority for island and reef management.

**Supplementary Information:**

The online version contains supplementary material available at 10.1038/s41598-024-76759-2.

## Introduction

Anthropogenic nutrient input from pollution and agricultural runoff has long been known to negatively affect coral reef health by decreasing coral growth rates and by increasing the abundance of phytoplankton, weedy algal species, and filter feeders (e.g., reviewed in^1,2^). Natural nutrient subsidies, on the other hand, can benefit coral reef ecosystems by supplying balanced nutrients to an otherwise nutrient-limited system^3,4^. Local sources of natural nutrients include fish aggregations^5,6^ or seabird colonies on nearby islands^7,8^. Seabirds effectively concentrate large amounts of nutrients from their open ocean prey onto small tropical islands through guano deposits^9–11^. These nutrient subsidies leach into adjacent marine ecosystems and are assimilated by primary and secondary producers up to at least 100–300 m offshore, traceable through elevated δ^15^N signals in corals, sponges, macroalgae and herbivorous fish^8,12,13^.

Corals assimilate nutrients through their symbiotic algae and can use the additional energy from nutrient-subsidies for growth^4^. Branching *Acropora* corals next to seabird islands have shown to grow 4-times faster (linear growth rate^14^) or 2-times faster (planar area increase^15^) compared to islands with few seabirds. These growth metrics are easy to determine for branching coral species and indicate changes in overall space occupancy^16^. However, it is not yet known if seabird-derived nutrients also enhance the growth of other coral genera and slower-growing morphologies. Also, it is unclear if the same effect is visible for more meaningful colony-scale growth parameters (surface area increase, volume increase), which are for instance used to normalize physiological processes to colony size^17^ and to calculate calcification rates. Calcification rates are the most comparable measure of coral growth^16^ and a more relevant metric to estimate geo-ecological reef functions^18^. Geo-ecological reef functions include reef framework production and vertical reef accretion, which are essential to provide habitat to marine species, protect coasts and islands from wave energy, and to keep up with sea-level rise^19^. The ability of a reef to provide these functions is indicated by its carbonate budget, which can be quantified using the survey-based ReefBudget methodology^20^.

The remote Chagos Archipelago has been used as a natural laboratory for many of the existing studies on seabird nutrient impacts on reefs, as it harbours islands with varying seabird densities^21^ and is not exposed to any local pollution^22^. Seabird densities on these islands are controlled by invasive rats, which predate on bird eggs and chicks^23,24^ and decimated seabird populations on some, but not all the islands in the Archipelago. This study compared coral growth between reefs adjacent to an island with high seabird densities and a nearby island with very few seabirds. We quantified a range of colony-scale growth metrics (linear extension, planar area increase, surface area increase, volume increase) for the submassive coral *Isopora palifera* and the compact branching (corymbose) coral *Acropora vermiculata* using photogrammetry and 3D modelling (*I. palifera*) or planar and side-view photographs (*A. vermiculata*). Volume increase was multiplied by skeletal bulk densities of coral colony fragments to calculate calcification rates. Subsequently, site-specific coral growth and calcification rates were integrated with benthic ReefBudget surveys^20^ to test whether seabird presence influences reef-scale carbonate production. We hypothesized that both colony- and reef-scale calcification rates will benefit from seabird-derived nutrient subsidies.

## Results

### Coral colony growth

**Fig. 1:**
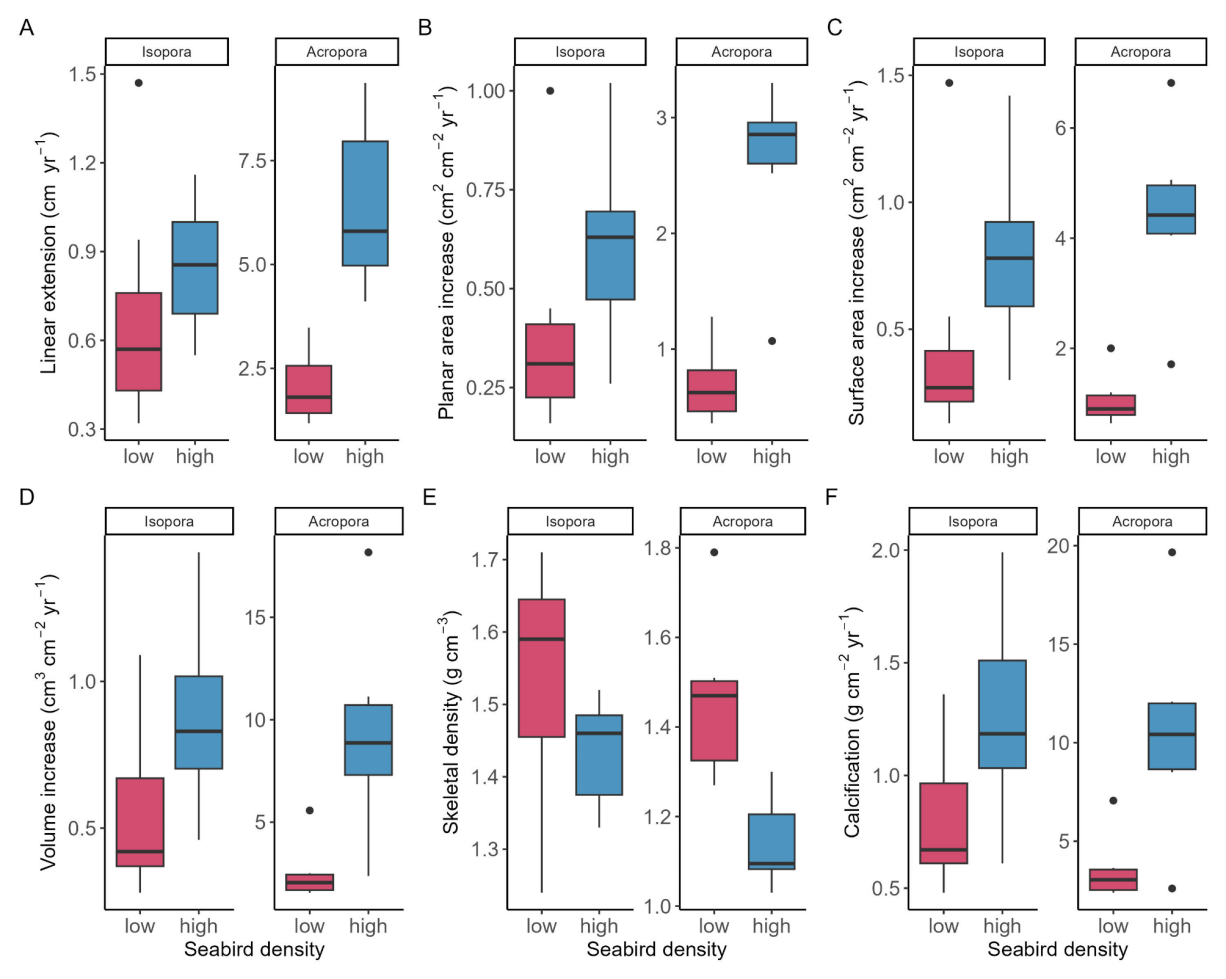
Colony-scale growth metrics for submassive *Isopora palifera* and corymbose *Acropora vermiculata* next to an island with low seabird densities (low) and an island with high seabird densities (high). (**A**) linear extension rate (cm yr^− 1^), (**B**) planar area increase (cm^2^ cm^− 2^ yr^− 1^), (**C**) surface area increase (cm^2^ cm^− 2^ yr^− 1^), (**D**) volume increase (cm^3^ cm^− 2^ yr^− 1^), (**E**) skeletal bulk density (g cm^− 3^), and (**F**) calcification rate (g CaCO_3_ cm^− 2^ yr^− 1^). All metrics except **A** and **E** are normalized to initial surface area of each colony. Boxes represent the median and the interquartile range (IQR), with whiskers extending to 1.5 times the IQR and outliers represented by points.

Submassive *I. palifera* colonies close to the island with high seabird densities displayed 1.4-times higher median linear extension rates (95% highest posterior density interval [95% HPDI] = 0.8–2.1), 1.8-times faster planar area increase (0.8-3.0), 2.1-times faster surface area increase (0.8–4.1), and 1.7-times faster volume increase (1.0-2.3) compared to colonies close to the island with low seabird densities (Fig. 1A-D). Corymbose *A. vermiculata* colonies close to the seabird island displayed 3.2-times higher linear extension rates (1.8–5.1), 3.9-times faster planar area increase (1.9–6.7), 4.1-times faster surface area increase (2.1-7.0) and 3.5 -times faster volume increase (1.3–6.8) compared to the island with few seabirds. Skeletal bulk densities correlated negatively with linear and planar extension rates for both coral species (Figure S1). However, despite 1.1- and 1.3-times higher skeletal bulk densities at the low seabird island ([1.0-1.2] and [1.1–1.5], respectively) (Fig. 1E), median calcification rates were 1.6 -times higher at the island with high seabird densities for *I. palifera* (1.0-2.3) and 2.7 -times higher for *A. vermiculata* (1.0-5.1) (Fig. 1F). Estimated seabird effects on all growth metrics and posterior probabilities are also displayed in Figure S2 and Table S1.

### Reef-scale carbonate production

**Fig. 2:**
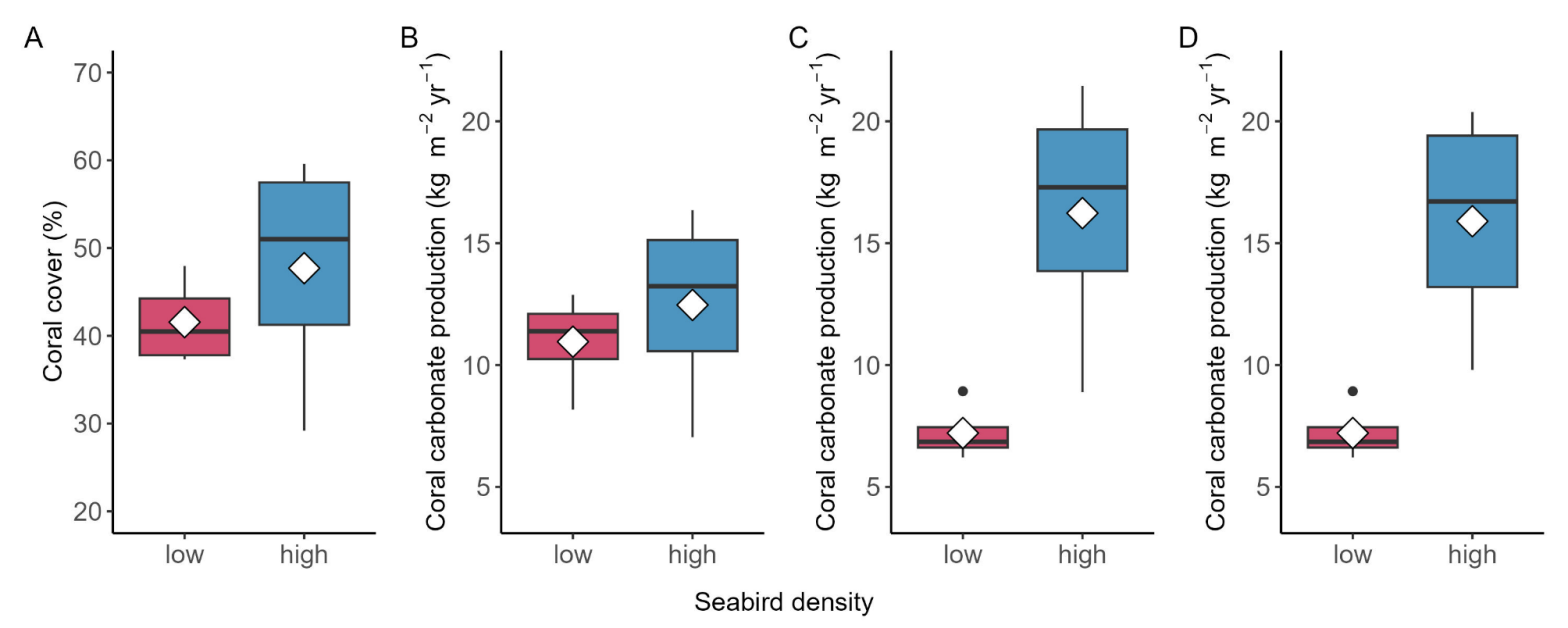
Coral cover and reef-scale coral carbonate production rates. (**A**) Coral cover (%); Reef-scale coral carbonate production calculated (**B**) using average coral growth rates and skeletal densities at both sites, (**C**) using site-specific linear growth rates and skeletal density values for submassive *Isopora palifera* and corymbose *Acropora vermiculata*, and (**D**) using the same coral growth rates for both sites (as in **C** low seabird) but multiplying carbonate production at the high seabird site with 2.2 (mean difference in calcification across both species). Boxes represent the median and the interquartile range (IQR), with whiskers extending to 1.5 times the IQR and outliers represented by points. White diamonds indicate the mean values for each group.

Total hard coral cover was similar at both sites (mean ± SE: 42 ± 2 and 48 ± 7%; Fig. 2A), while cover of corymbose *Acropora* was considerably lower at the island with low seabird densities (26 ± 6% compared to 41 ± 8%). The next most common taxa at the island with low and high seabird density, respectively, were submassive *Isopora* (5 ± 2 and 1 ± 1%), massive *Favites* (5 ± 1 and 2 ± 1%), tabular *Acropora* (2 ± 2 and 0%), massive *Porites* (1 ± 1 and 2 ± 1%) and different encrusting coral taxa (3 ± 1 and 1 ± 1%). To illustrate the importance of using site-specific coral growth data, reef-scale carbonate production was calculated from benthic cover data using three different methods – applying average regional growth rates at both sites^25^, applying site-specific coral growth rates for *Acropora* and *Isopora* (from this study), and using the mean difference in calcification rates as a multiplication factor (from this study). When applying the same regional coral growth rates and skeletal densities, total coral carbonate production was similar at both islands (median and [95% HPDI]: low seabird = 10.8 [6.8–15.6], high seabird = 11.9 [7.5–17.5] kg m^− 2^ yr^− 1^; Fig. 2B). However, when using the site-specific linear growth rates and skeletal densities of *A. vermiculata* and *I. palifera* for calculations, total coral carbonate production next to the island with high seabird densities was 2.2-fold higher compared to the island with low seabird densities (1.0–3.6) (low seabird = 7.1 [4.3–10.3], high seabird = 15.5 [9.7–22.6] kg m^− 2^ yr^− 1^; Fig. 2C). In this case, *Acropora* and *Isopora* contributed 66 and 14% of total carbonate production at the island with low seabird densities and 92 and 1% at the island with high seabird densities, respectively. A similar difference in total coral carbonate production was visible when, instead of using site-specific coral growth rates, coral carbonate production calculated with the same growth rates was multiplied by 2.2 (mean difference in calcification across both species) at the island with high seabird densities (15.2 [10.4–20.9] kg m^− 2^ yr^− 1^; Fig. 2D).

## Discussion

This is the first study to show that seabird-derived nutrient subsidies increase growth and calcification rates of multiple coral taxa, which in turn affects reef-scale carbonate production. These results emphasize that seabirds can directly influence important reef ecosystem functions.

Enhanced coral growth in response to artificial but balanced nutrient addition^26,27^ or natural seabird-derived nutrient subsidies^14,15^ has been shown previously for corals of the genus *Acropora*. As *Acropora* spp. are among the fastest growing coral species^16^ and show distinct responses to nutrient treatments^27^, it was thus far unknown if seabird-nutrient benefits would extend to other coral genera as well. Here, we show that seabird nutrients also enhance growth rates of an *Isopora* coral, suggesting that natural nutrient additions benefit the growth of several coral taxa, including submassive and branching coral species. Effect sizes were higher for *A. vermiculata* compared to *I. palifera* for all growth metrics. It is possible that different methods used to estimate growth contributed to this difference in effect size (top-down photographs for *A. vermiculata* versus 3D photogrammetry for *I. palifera*). However, the higher uncertainty for *A. vermiculata* estimates would be expected to decrease rather than increase the effect size for these corals. Thus, it is much more likely that higher overall growth rates^16^ of *A. vermiculata* or increased ability to uptake and utilize nutrients compared to other corals^4,28^ explain the stronger effects of seabird nutrients on this species compared to *I. palifera.* Nutrient effects on planar area increase of *A. vermiculata* in this study (4-fold) were higher than reported previously for the Chagos Archipelago (2-fold^15^). Contrary to our study, which focused on Salomon atoll where the shallow enclosed lagoon promotes high retention of nutrients and thereby maximises nutrient effects^29^, the previous study integrated several larger atolls, explaining the difference in effect size. Also, the previous study found that growth rates increased over time (2018–2021), and by only using the latest timepoint for this study (2020–2021) we can be certain that corals recovered fully from the bleaching event in 2015–2016 and potential transplantation stress in 2018^15^. Despite a relatively small sample size from only two sites, our results align well with the overall pattern of nutrient effects in the Chagos Archipelago, and the 3- to 4-fold increase in *Acropora vermiculata* growth metrics matches seabird nutrient effects on *Acropora formosa* linear extension in Fiji^14^. It is unlikely that other sources of reef variability impacted growth rates, as the close proximity of islands facilitates similar physico-chemical reef environments (e.g. wave energy, water temperature, etc.)^30^ aside from the difference in seabird nutrients. The presence of seabirds however was shown to cause enriched nutrient values in reef algae^31^ and coral algal symbionts^15^ at the studied reefs.

In addition to documenting the effects of seabird-derived nutrients on a coral taxon other than *Acropora*, this study for the first time examined multiple growth metrics. It was previously unknown if the observed increases in linear^14^ or planar extension^15^ would translate to increased colony-scale calcification rates, due to the typically inverse relationship of skeletal density and growth rates^16^. While linear and planar extension are important indicators of space occupancy, they disregard vertical colony growth and are not comparable among different coral morphologies^16^. Coral calcification is the most important metric underpinning reef geo-ecological functions^19^ and our study shows that despite reduced skeletal density in faster growing corals, colony-scale calcification rates were 2 to 3-times higher where seabird nutrients were present. On a reef-scale, carbonate production supports reef framework production and vertical reef accretion, which is essential to provide habitat to marine life, to protect adjacent coasts from storms and erosion, and to keep-up with sea-level rise^19^. Management actions often aim to increase coral cover but fail to look at these important ecosystem functions. While higher coral cover generally translates to higher carbonate production^32^, we found that a detailed assessment of site-specific coral growth rates is necessary to accurately evaluate differences in ecosystem functions where environmental conditions vary across sites. Specifically, coral cover was similar at both sites, but reef-scale carbonate production was > 2-fold higher close to the seabird-rich island when using site-specific growth rates. Using the same growth rates as both sites but multiplying carbonate production by 2.2 (which is the average multiplication factor of calcification increases across both species) at the high seabird site yielded the same results. This suggests that using a multiplication factor might be an easy way of integrating nutrient effects on carbonate production estimates if site-specific growth rates are not available. Of course, this ratio would vary with coral composition and levels of nutrient input, so an extrapolation to other reefs should be carefully considered.

The fact that increased coral growth at the seabird-rich island does not lead to significantly higher coral cover indicates that the overall turnover of calcium carbonate may be higher. Indeed, higher reef-scale erosion rates were found on nutrient-rich reefs caused by higher abundance and biomass of bioeroding parrotfishes^8^. However, despite limited effects of seabird nutrients on overall coral cover (also see^15,33^), coral recovery after large-scale mass bleaching was modelled to be faster on reefs close to seabird-rich islands^15^, indicating that after stress events the ecosystem benefits from natural nutrient pathways. Our results add to the growing pool of evidence that natural, well-balanced nutrients have very different effects than anthropogenic nutrients^3,34^, and that natural nutrient flows from seabirds benefit coral reef ecosystem functions^8,15,35^. Restoring natural nutrient pathways should thus be considered as a priority for island and reef management.

## Methods

### Study sites

Salomon atoll is the smallest of the northern atolls in the remote Chagos Archipelago, central Indian Ocean. Nine low-lying sandy islands and a reef flat encircle a shallow protected lagoon (24 km^2^, mean depth 17 m^29^), with an opening at the northern side of the atoll. Three of these islands harbour seabird colonies, while the others have hardly any seabirds due to the presence of invasive rats^36^.

Coral growth rates and reef-scale carbonate production was quantified on lagoonal reefs (1.5 m depth) adjacent to one island with high seabird densities and high nutrient input (Ile de la Passe, 19 breeding pairs ha^− 1^, 6.1 kg N input ha^− 1^ yr^− 1^) and one rat-infested island with very few seabirds and low nutrient input (Ile Anglaise, 0.6 breeding pairs ha^− 1^, 0.1 kg N input ha^− 1^ yr^− 1^)^21,36^. The islands are both situated along the Northwestern side of Salomon Atoll, approximately 4 km apart, and the lagoonal reefs therefore experience similar hydrodynamic and physico-chemical environmental conditions (wind direction, wave energy, tide range, water residence time, net primary productivity, temperature, salinity, oxygen)^29,30^. Genetic variability among islands is unlikely due to the close proximity of sites and generally high coral connectivity within the Chagos Archipelago^37^. Furthermore, a previous reciprocal transplant experiment between islands showed *Acropora* growth differences were caused by seabird nutrients, rather than coral origin (i.e., corals transplanted to high seabird density islands had similar growth rates, regardless of source island)^15^.

### Coral colony growth

Coral colonies were tagged and photographed over two consecutive years to estimate colony-scale growth metrics. Submassive *I. palifera* colonies were tagged and photographed in January 2022 (*n* = 9 at each site) and relocated and photographed in November 2023 (*n* = 8 at Ile de la Passe, *n* = 7 at Ile Anglaise, *n* = 3 dead or lost). Corymbose *A. vermiculata* colonies at the same sites were monitored for a different experiment (2018-2021^15^), and photos taken of the same colonies in 2020 and 2021 were re-analysed for this study (*n* = 6 colonies at each site). Besides being closest to the observation time for *I. palifera* colonies, using the latest time interval ensured that corals recovered fully from the bleaching event in 2015–2016 and potential transplantation stress in 2018^15^.

*I. palifera* colonies were 3D modelled from photographs taken from all angles (*n* = 30–50 photos per colony) using Agisoft Metashape Professional (v1.8.4) and aligned in CloudCompare^38^. A variety of growth metrics (average linear extension rate, planar area increase, surface area increase, volume increase) were calculated from the comparison of 3D models^39^. Linear extension rates present average growth over the entire colony as typically extracted for submassive/massive coral morphologies^39^. Maximum linear extension rates at branch tips can be found in the raw data table. For *A. vermiculata* colonies, not enough photographs were available for 3D modelling. Instead, top-down photographs were used to extract the planar area and maximum diameter for each colony and year using ImageJ/FIJI^15^ and side-view photos with a reference scale were used to determine colony height. The difference in maximum diameter between years was divided by 2 to yield linear extension rates. Colony surface area was calculated from maximum diameter (D_max_) using a power relationship modelled specifically for densely branching *Acropora* (Surface area = 0.837*D_max_^2.459^  Chandler et al.^40^). Volume was calculated by multiplying planar area with colony height. Due to geometric approximation of *Acropora* colony surface area and volume, a higher uncertainty is associated with these metrics, although relative differences between the sites are considered reliable due to the use of consistent calculation methods.

Additionally, a small fragment (~ 4–6 cm) was sampled from each coral colony at the end of the study to determine skeletal bulk density by buoyant-weighing wax dipped fragments^41^. Skeletal bulk density was then multiplied with volume increase to calculate calcification rate. Growth metrics, except linear extension rates, were normalized to initial surface area of coral colonies to ensure comparability of results across colonies of different sizes, and all metrics were converted to annual rates.

### Reef-scale carbonate production

Reef-scale carbonate production was estimated using the ReefBudget methodology^20^, which quantifies the balance between calcium carbonate (CaCO_3_) production and bioerosion on a reef using survey data of calcifying and bioeroding organisms and species-specific calcification/bioerosion rates (method handbook and calculation sheets at https://geography.exeter.ac.uk/reefbudget/). We only compared coral carbonate production for this study as we were primarily interested in the effects of nutrient subsidies on coral growth. At each study island, coral colony sizes were measured to the nearest cm along the 3D reef contour under four replicate transects (10 m) in November 2023 and combined with taxa-specific linear growth rates and skeletal densities to yield reef-scale coral carbonate production. To illustrate the importance of using site-specific growth data for carbonate production estimates we compared three different scenarios: (A) Calculations for both sites used Chagos-specific calcification rates for 20 coral genera^25^ and averages over Indo-Pacific rates for the remaining coral taxa; (B) Linear growth rates and skeletal density values for *Isopora* and corymbose *Acropora * were replaced with site-specific values recorded for *I. palifera* and *A. vermiculata* in this study; and (C) Calculations for both sites used growth rates at the low seabird island, but resulting carbonate production at the island with high seabird densities was multiplied by 2.2 to reflect average increases in coral calcification rates compared to the seabird-poor site as found in this study.

### Statistical analysis

Differences in colony growth metrics and carbonate budgets between sites were tested using Bayesian models. Bayesian models can perform better than frequentist methods with small sample sizes but can be sensitive to prior specifications^42^. To be conservative, we used the recommended half-Cauchy variance prior and default (weakly uninformative) priors for the seabird parameter (as no prior data exists for the effect of seabirds on *Isopora* coral, most growth metrics of *Acropora*, or reef-scale carbonate production rates). Models were run on log-transformed response parameters to enable easy comparison of seabird effects on different growth metrics and taxa (log(growth metric/carbonate production) ~ seabird density). Values are reported as median and 95% highest posterior density interval (HPDI), which is defined as the narrowest interval containing the specified probability mass. All models were run for four chains, with 3,000 iterations and 1,000 warm-up iterations per chain, and checked for model convergence and fit using posterior predictive checks, traceplots, and the Gelman-Ruban convergence diagnostic (R-hat)^43^. All analyses were conducted in R 4.2.0^44^ and Bayesian models were implemented in STAN using the brms package^45,46^, along with the tidybayes^47^ and emmeans^48^ packages.

## Electronic supplementary material

Below is the link to the electronic supplementary material.

Supplementary Material 1

## Data Availability

Data files and R code for analysis can be found at https://github.com/InesLange/seabird-nutrients-coral-growth.

## Declarations

*Open access statement*.
