# Supplementary material for: Seabird nutrients increase coral calcification rates and boost reef carbonate production
Source: Sci Rep. 2024 Oct 22;14:24937. doi: 10.1038/s41598-024-76759-2 (PMC11496823; doi:10.1038/s41598-024-76759-2)
Supplement: Supplementary file 1 — Supplementary Material 1 [file 41598_2024_76759_MOESM1_ESM.pdf]

Supplementary Material for:

Seabird nutrients increase coral calcification rates and boost reef carbonate production

Ines D Lange[1,*], Cassandra E Benkwitt[2]

[1] University of Exeter, Exeter, UK

[2] Lancaster Environment Centre, Lancaster University, Lancaster, UK

* Corresponding author i.lange@exeter.ac.uk

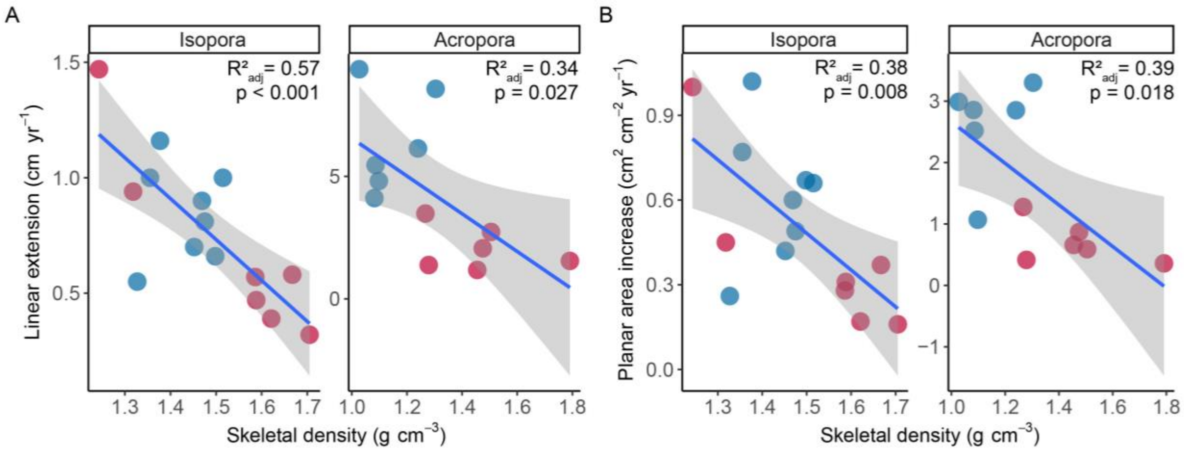

Figure S1: Relationship between skeletal density and coral growth. Skeletal density correlates negatively with linear growth rate (A) and planar area increase (B) for *Isopora palifera* and *Acropora vermiculata* (R^2_{adj} and p values displayed in plots). Data points are coloured according to seabird densities on adjacent islands, with colonies at the low seabird density/nutrient input site in red and colonies at the high seabird density site in blue.

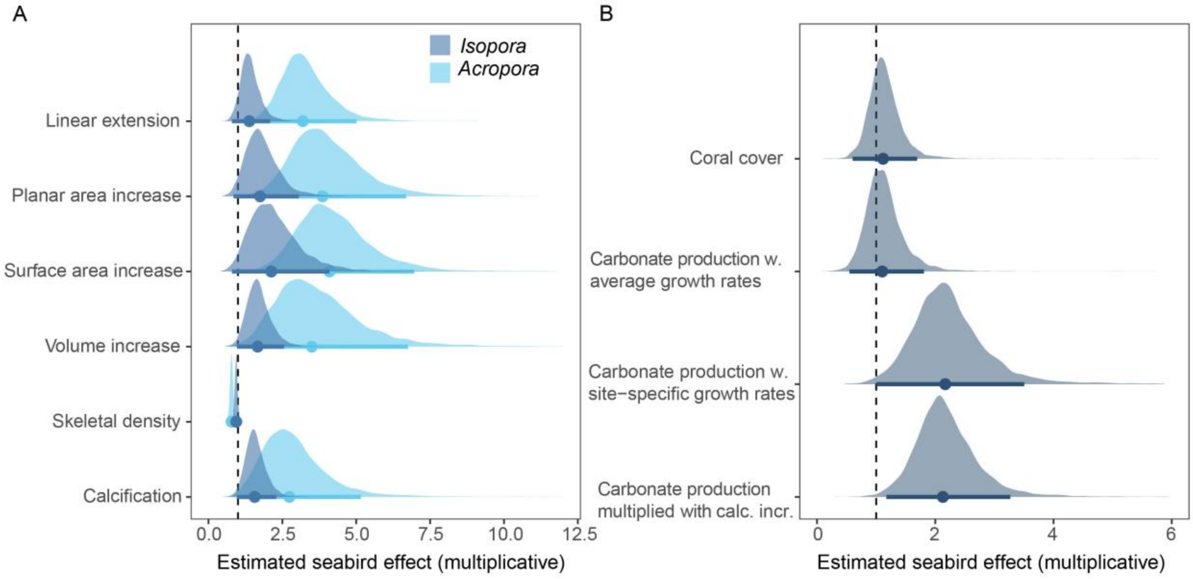

Figure S2: Estimated effect sizes of seabird-derived nutrients on coral growth and reef-scale carbonate production. A) Comparison of growth metrics for *Isopora palifera* (dark blue) and *Acropora vermiculata* (light blue) as derived from Bayesian models (log(growth metric) ~ seabird density). B) Comparison of reef-scale coral carbonate production as derived from Bayesian models (log(carbonate production) ~ seabird density). Shapes represent posterior distributions, points represent the median multiplicative change in growth at high seabird densities compared to low seabird densities and thick lines depict the 95% highest posterior density interval (HPDI).

Table S1: Results from Bayesian models testing the effect of seabird nutrients on coral growth metrics and reef-scale carbonate production. Median values [95% highest posterior density interval (HPDI)] for each category of seabird density (low, high), estimated median multiplicative effect of seabirds [95% HPDI], and posterior probability of a positive effect of seabirds on each metric (except density for which the effect is negative).

Metric	Median [95% HPDI]<br>Seabird density		Seabird effect (multiplicative) [95% HDPI]	Posterior probability of seabird effect
	low	high		
Isopora palifera				
Linear extension (cm yr^{-1})	0.60 [0.41 – 0.81]	0.83 [0.60 – 1.13]	1.38 [0.78 – 2.08]	0.92
Planar area increase (cm^2 cm^{-2} yr^{-1})	0.33 [0.19 – 0.48]	0.57 [0.36 – 0.84]	1.75 [0.80 – 3.01]	0.96
Surface area increase (cm^2 cm^{-2} yr^{-1})	0.32 [0.16 – 0.52]	0.68 [0.35 – 1.08]	2.13 [0.79 – 4.10]	0.97
Volume increase (cm^3 cm^{-2} yr^{-1})	0.50 [0.35 – 0.69]	0.83 [0.57 – 1.11]	1.66 [0.98 – 2.57]	0.98
Skeletal density (g cm^{-3})	1.52 [1.41 – 1.64]	1.43 [1.33 – 1.54]	0.94 [0.84 – 1.04]	0.88 (neg effect)
Calcification rate (g cm^{-2} yr^{-1})	0.76 [0.55 – 1.02]	1.19 [0.88 – 1.57]	1.56 [0.96 – 2.29]	0.98
Acropora vermiculata				
Linear extension (cm yr^{-1})	1.92 [1.28 – 2.69]	6.14 [4.05 – 8.42]	3.19 1.80 – 5.05	>0.99
Planar area increase (cm^2 cm^{-2} yr^{-1})	0.63 [0.39 – 0.95]	2.46 [1.48 – 3.58]	3.86 1.90 – 6.71	>0.99
Surface area increase (cm^2 cm^{-2} yr^{-1})	0.99 [0.63 – 1.47]	4.08 [2.60 – 5.99]	4.10 [2.08 – 7.00]	>0.99
Volume increase (cm^3 cm^{-2} yr^{-1})	2.28 [1.15 – 3.70]	7.98 [4.25 – 13.40]	3.50 [1.28 – 6.81]	>0.99
Skeletal density (g cm^{-3})	1.45 [1.29 – 1.60]	1.14 [1.02 – 1.26]	0.78 [0.67 – 0.90]	>0.99 (neg effect)
Calcification rate (g cm^{-2} yr^{-1})	3.33 [1.72 – 5.28]	9.10 [4.60 – 14.40]	2.74 [0.99 – 5.11]	>0.99
**Reef-scale coral carbonate production**				
Coral cover (%)	41.2 [28.3 – 56.2]	46.1 [31.4 – 62.9]	1.12 [0.63 – 1.72]	0.71
Carbonate production with average growth rates (kg m^{-2} yr^{-1})	10.8 [6.83 – 15.6]	11.9 [7.51 – 17.5]	1.10 [0.55 – 1.81]	0.65
Carbonate production with site-specific growth rates (kg m^{-2} yr^{-1})	7.12 [4.34 – 10.30]	15.47 [9.65 – 22.60]	2.17 [0.99 – 3.58]	0.99
Carbonate production with multiplication factor 2.2 (kg m^{-2} yr^{-1})	7.13 [4.86 – 9.84]	15.22 [10.4 – 20.94]	2.13 [1.17 - 3.28]	>0.99
